# Effect of densification pretreatment on combustion and particulate matter emission characteristics of agricultural biomass

**DOI:** 10.1098/rsos.240848

**Published:** 2025-04-02

**Authors:** Wei Yang, Shilong Feng, Yongming Xu, Youjian Zhu, Shanzhi Xin, Wenbo Hu, Heyong Li, Pan Li, Huihui Liu, Haiping Yang

**Affiliations:** ^1^ Zhengzhou University of Light Industry, Zhengzhou, Henan, People’s Republic of China; ^2^ Technology Center, China Tobacco Henan Industrial Co., Ltd., Zhengzhou, Henan, People’s Republic of China; ^3^ Jianghan University, Wuhan, Hubei, People’s Republic of China; ^4^ Zhengzhou University, Zhengzhou, Henan, People’s Republic of China; ^5^ Huazhong University of Science and Technology, Wuhan, Hubei, People’s Republic of China

**Keywords:** biomass, combustion, densification, particulate matter

## Abstract

Agricultural biomass production in China is substantial, and the densification pretreatment of agricultural biomass has the potential to reduce usage costs and increase fuel energy density. However, there is still uncertainty regarding the impact of densification pretreatment on combustion characteristics and particulate matter emissions. In this study, the combustion characteristics of raw biomass and biomass pellets were investigated using a thermogravimetric analyser and fixed-bed reactor with cotton stalk and rice husk. The findings indicate that biomass densification pretreatment enhances combustion performance, resulting in more concentrated and intense combustion compared with untreated biomass. The combustion models become more intricate due to the process of densification pretreatment. For cotton stalk, densification pretreatment proves beneficial in reducing the emission of PM_0.1–10_ (particulate matter with a diameter between 0.1 and 10 µm), although it does not exhibit a discernible inhibitory effect on ultrafine particles (<0.1 μm). Densification pretreatment has also been shown to suppress the conversion of alkali metal sulfides into particulate matter. In the case of rice husk, densification pretreatment primarily reduces PM_1–10_ emissions while simultaneously increasing the formation of fine particles (<1 μm). This process facilitates the interaction between Si and alkali metal chlorides, thereby enhancing silicate reaction and impeding the conversion of alkali metal chlorides into PM_1_. However, the impact of densification pretreatment on the elemental composition of PM_1–10_ remains insignificant in both biomass samples.

## 1. Introduction

As the world’s largest developing country, China already tops the list of annual carbon emissions. The Chinese government aims to reduce carbon emissions through various initiatives and eventually achieve carbon neutrality by 2060 [1]. As a major agricultural country, China has a huge agricultural biomass output, with an annual output of 829 million tons, of which approximately 694 million tons can be collected; however, the energy utilization rate is only 10.6% [2]. In response, the Chinese government had issued a series of policy documents to promote the reuse of agricultural biomass resources. In March 2023, the National Energy Administration, the Ministry of Ecology and Environment and other government departments recommended to promote the development of biomass industries, such as biomass pellets and biomass boilers. In April 2024, the National Development and Reform Commission proposed reducing carbon emissions by relying on technologies, such as biomass energy utilization and agricultural and forestry waste resource utilization.

In recent years, the enormous potential of biomass in the energy field has received much attention. Among numerous biomass utilization technologies, biomass combustion for heating and power generation is one of the most efficient and feasible solutions [3]. Biomass can be applied to various combustion devices, such as power plant boilers, steam boilers, household heating stoves based on size and fuel characteristics [4,5]. The biomass usage proportion in renewable energy generation in the UK has reached 30.8%, playing a pivotal role in economic development. To achieve energy conservation, emission reduction and sustainable development, other European countries such as Denmark, Finland and France are planning to gradually replace coal with biomass [6]. Sweden has a unique advantage in the field of biomass pellet industry, occupying a large European market with the help of pellet standards (ENplus) [7]. Seven of the world’s top 10 pellet manufacturers are located in Europe, including Sweden, Germany, Latvia and others [8]. Savolainen [9] conducted mixed combustion tests of biomass and coal in commercial boilers, demonstrating the advantages of biomass in reducing CO_2_, SO_2_ and NO_*x*_ emissions. Li *et al*. [10] performed mixed volumetric combustion of biomass and coal to reduce CO_2_ and NO emissions. Additionally, Allgurén *et al*. [11] found that biomass and coal mixing can significantly reduce NO emission. Wang *et al*. [12] reduced SO_2_ emission through mixed combustion of biomass and coal. Nunes *et al*. [13] posited that the demand for biomass in the energy sector would certainly escalate, with agricultural waste emerging as a pivotal energy source.

The utilization of agricultural biomass requires comprehensive consideration of application scenarios, operability, cost and other factors. Compared with other biomass such as wood, agricultural biomass has low calorific value, low density and high cellulose content. These characteristics make continuous feeding difficult during the application process and limit the utilization scale [14]. To improve the utilization efficiency of agricultural biomass and reduce usage cost, various pretreatment technologies have been proposed, including physical (size reduction, drying, milling and densification), thermal (torrefaction and steam explosion) and chemical pretreatments (acid or alkali) [15]. Size reduction, drying and milling are basic pretreatment methods to reduce biomass moisture content and increase the convenience of transportation and feeding. However, the density of broken agricultural biomass remains low, with high transportation cost and easy water absorption. Therefore, thermal pretreatment is used to further improve biomass fuel characteristics. Torrefaction removes hydrophilic components from agricultural biomass [16]. Torrefaction can increase the calorific value of agricultural biomass, and enhance its thermal stability and hydrophobicity [17]. For biomass combustion, torrefaction is beneficial in reducing sulfur oxide and nitrogen oxide emissions [18]. Similar to torrefaction, steam explosion obtains high-quality (in terms of mechanical strength, energy density, water resistance, among others) pellet products by improving the internal biomass structure. Steam explosion has good applicability for lignocellulosic biomass, but the applicability for agricultural biomass is unknown [19]. Thermal pretreatment requires a high energy input, which increases pretreatment costs. Chemical pretreatment involves various acid or alkali solutions, which not only alter the internal biomass structure but also remove specific elements too [20]. The corrosiveness offered by the chemical pretreatment hinders its commercial application [21,22].

Densification pretreatment is another method that has gained attention due to its ability to improve the convenience of biomass utilization [23]. Compared with the aforementioned pretreatment techniques, densification pretreatment is advantageous as it produces biomass fuel with uniform properties and high energy density, thereby reducing any transportation hazard [24]. Besides, the densification pretreatment cost is lower than that of thermal and chemical pretreatments. Due to the uniform shape and properties of densified biomass, it can be used in various commercial kilns [25]. Muazu *et al*. [26] evaluated five biomass densification pretreatment cases and found that the technology effectively reduces energy loss and greenhouse gas emissions. Guo *et al*. [27] found that densification pretreatment reduced sulfur and nitrogen emissions.

Despite these advantages, biomass combustion still faces the problem of other pollutant emissions, especially particulate matter emissions. In Europe, biomass combustion emissions account for only 1.9% of that of total primary energy; however, PM_2.5_ (particulate matter with an aerodynamic diameter of less than 2.5 µm) emissions are as high as 36% [28]. Feldmeier *et al*. [29] found that particulate matter produced by burning agricultural biomass was significantly higher than that produced by wood biomass. Therefore, particulate matter emissions from agricultural waste utilization deserve much attention. Han *et al*. suggested that torrefaction reduced the alkali metal content in biomass particles, leading to suppression of particulate matter emissions [30]. Cheng *et al*. [31] further indicated that torrefaction can promote the reaction of alkali metals and alkaline earth metals (AAEMs) with silicates and aluminosilicates, achieving the conversion of fine particles to coarse particles. These studies demonstrated that torrefaction has been effective in reducing particulate matter emissions. While the influence of densification pretreatment on particulate matter emission remains yet to be studied, densification pretreatment has an inhibitory effect on nitrogen oxide and sulfur oxide emissions. Studies on particulate matter emission are crucial for comprehensive understanding of the effect of densification pretreatment on pollutant emissions from biomass combustion. Additionally, these studies can promote technology development for clean utilization of biomass.

In this study, two typical local agricultural biomass in China, namely cotton stalk and rice husk, were selected due to their large annual yields [32,33]. The influence of densification pretreatment on the combustion process and mechanism was studied. The emission and composition of particulate matter after densification pretreatment were analysed. The comprehensive study of combustion processes and particulate matter emission characteristics will contribute to efficient utilization of agricultural biomass.

## 2. Material and methods

### 2.1. Fuel properties and ash composition

Two typical agricultural biomass, namely cotton stalk and rice husk, were selected for combustion. These biomass samples were crushed into a powder form with a particle size of less than 0.2 µm. To determine moisture content, the samples were placed in an oven at 55°C for at least 4 h until no more weight loss is observed. The samples were subsequently kept in a 105°C oven to remove the moisture content for proximate and ultimate analysis. As shown in table 1, the ash content of rice husk is 16.20%, which is much higher than that of cotton stalk (2.74%), whereas the volatile and fixed carbon content of cotton stalk is higher than that of rice husk. In addition, the C, H, N, S content and lower heating value of cotton stalk are higher than those of rice husk.

**Table 1.** Fuel properties and ash composition.

samples	cotton stalk	rice husk
proximate analysis (wt%)		
*M*_ad_	4.55	3.31
A_db_	2.74	16.20
*V*_db_	78.61	69.12
FC_db_	18.65	14.69
ultimate analysis (wt%)		
C_db_	45.72	38.2
H_db_	5.43	3.15
N_db_	1.09	0.89
S_db_	0.24	0.19
O_db_	44.77	41.38
LHV (MJ kg^−1^)	15.97	11.66
ash composition (wt%)		
MgO	9.06	—
Al_2_O_3_	2.24	—
SiO_2_	3.87	96.36
P_2_O_5_	11.59	—
SO_3_	11.74	0.55
Cl_2_O	2.37	—
K_2_O	30.51	1.80
CaO	27.94	0.88
Fe_2_O_3_	0.58	0.24

–, not detected; A, ash; ad, air dried basis; db, dried basis; FC, fixed carbon; M, moisture; V, volatile.

The two samples were placed in a muffle furnace and burned for 4 h at 600°C to obtain ashes, which were then analysed using X-ray fluorescence spectroscopy. As shown in table 1, cotton stalk ash was mainly composed of AAEMs. Unlike cotton stalk ash, rice husk ash was mainly composed of silica.

### 2.2. Combustion experiment

Combustion characteristics of the two samples were analysed by thermogravimetric analyser. The reaction atmosphere was supplied with nitrogen and oxygen at a ratio of 80 : 20, with a total gas flow rate of 100 ml min^−1^. The reaction temperature ranged from 25°C to 900°C, with a heating rate of 10°C min^−1^. A set of blank experiments was done before placing the samples to facilitate experimental errors correction. Four types of samples, namely raw cotton stalk (RCS), cotton stalk pellet (CSP), raw rice husk (RRH) and rice husk pellet (RHP), were used. The RCS and RRH refer to the powder samples with a particle size less than 0.2 µm, whereas CSP and RHP are cylindrical particles with a diameter of 6 mm and varied length (based on sample mass) prepared in the laboratory. Biomass samples were densified into pellets on a universal material testing machine (WDW3200, Kexin Corporation, China). The sample (0.1 g) was weighed and put into the extrusion sleeve for densification. During compression, the piston rod moved at a speed of 20 mm min^−1^ until the specified pressure (7000 N) was reached, and the compression stopped and remained for 2 min. The pellet was squeezed out at the other side of the mould at 5 mm min^−1^. The detailed densification process of biomass pellets can be found in previous literature [34].

### 2.3. Particulate matter collection

The particulate matter collection during biomass combustion was conducted in a one-dimensional vertical reaction furnace. The reactor was composed of a feeding system, electric heating device, gas cylinder, gas flow meter, cyclone separator, cooling air duct, particulate matter collection device and vacuum pump. The combustion temperature was set at 850°C, and the air atmosphere was simulated using high-purity nitrogen and oxygen. The samples were transported from the feeding device situated at the top of the reactor, with 2.5 l min^−1^ of airflow falling down to the middle stainless steel baffle for complete combustion. One biomass pellet (0.1 g) per min was added to the reactor through the star valve feeder for a total of 20 min. High-temperature flue gas flowed from the bottom of the reactor, which got mixed with 7.5 l min^−1^ of cooling air in the cyclone separator. Particles less than 10 µm emerged from the cyclone and entered the particulate matter collection device, and were collected in 13 stages based on the aerodynamic diameter. Detailed structure and schematics can be found in previous studies [35].

### 2.4. Data processing and particulate matter analysis

To evaluate the combustion process, the ignition index (*D*_i_), burnout index (*D*_b_), flammability index (*F*) and comprehensive combustion index (*C*) are defined as follows [36]:


(2.1) $$D_{i}=\frac{{DTG}_{max}}{t_{i}\times t_{p}},$$

where DTG_max_ is maximum weight loss rate, *t*_i_ is ignition time and *t*_p_ is time corresponding to maximum weight loss rate.


(2.2) $$D_{b}=\frac{{DTG}_{max}}{\Delta t_{1/2}\times t_{i}\times t_{b}},$$

where$\;\Delta t_{1/2}\;$ represents the time range when DTG/DTG_max_ is equal to 0.5 and *t*_b_ represents burnout time.


(2.3) $$F=\frac{{DTG}_{max}}{T_{i}\times T_{i}},$$

(2.4) $$C=\frac{DTG_{max}\times DTG_{mean}}{T_{i}\times T_{i}\times T_{b}},$$

where *T*_i_ is ignition temperature and *T*_b_ is burnout temperature.

The kinetic analysis of the combustion process was carried out by non-isothermal method. The combustion process comprises simple thermochemical reactions. According to the Arrhenius equation and Coats–Redfern method, the reaction mechanism equation can be obtained as follows [37]:


(2.5) $$G\left(\alpha \right)=\frac{ART^{2}}{\beta E}exp\left(-\frac{E}{RT}\right),$$

where *G*(*α*) is the differential form of the most probable mechanism function of the reaction, and *α* is reaction conversion rate: $\alpha =(m_{0}-m)/(m_{0}-m_{\infty })$, $m_{0}$ and $m_{\infty }$ represent initial and final weight of the sample, respectively. *A* is a pre-exponential factor, *R* is a gas constant, *T* is temperature, β is heating rate and *E* is reaction activation energy. By taking logarithms on both sides of equations (2.5), (2.6) can be obtained as follows:


(2.6) $$ln\left[\frac{G\left(\alpha \right)}{T^{2}}\right]=\ln\left(\frac{AR}{\beta E}\right)-\frac{E}{RT}.$$

According to the reaction mechanism equation of G(α), the regression line of In [*G*(α)/*T*^2^] to 1 /*T* is obtained. The slope and intercept are obtained according to the fitting formula of the regression line equation, thus, the activation energy and pre-exponential factor of the reaction are calculated. The solid-state reaction kinetics model used is shown in table 2. Due to reaction complexity, it is necessary to use the Malek method to select the most reasonable mechanism equation. A standard curve is obtained by substituting the conversion rate *α*_i_ at different stages in equation (2.7)


(2.7) $$y\left(\alpha \right)=\frac{f\left(\alpha \right)\cdot g\left(\alpha \right)}{f\left(0.5\right)\cdot g\left(0.5\right)},$$

**Table 2.** The solid-state reaction kinetics models. Note: 1, 3/2, 2, 3 and 4 represent the reaction order corresponding to the reaction models, respectively.

models	reaction order	*f*(*α*)	*g*(*α*)
chemical reaction series model	F1	1*−α*	−ln(1−α)
	F3/2	(1*−α*)^1.5^	2(1*−α*)^−0.5^−2
	F2	(1*−α*)^2^	(1*−α*)^−1^
phase interface model	R1	1	*a*
	R2	2(1*−α*)^0.5^	1−(1*−α*)^0.5^
	R3	3(1*−α*)^1/3^	1−(1*−α*)^1/3^
diffusion mechanism model	D1	1/2*α*	*α* ^2^
	D2	[−ln(1*−α*)]^1/3^	(1*−α*)ln(1*−α*)+*α*
	D3	3(1*−α*)^2/3^/2[1−(1*−α*)^1/3^]	[1−(1*−α*)^1/3^]^2^
	D4	3/2[(1*−α*)^1/3^−1]	(1−2*α*/3)−(1*−α*)^2/3^
random nucleation model	A2	2(1*−α*)[−ln(1*−α*)]^1/2^	[−ln(1*−α*)]^1/2^
	A3	3(1−*α*)[−ln(1−*α*)]^2/3^	[−ln(1*−α*)]^1/3^

A, random nucleation model; D, diffusion mechanism model; F, chemical reaction series model; R, phase interface model.

where y(α) is standard curve function, and f(α) and g(α) represent the differential and integral forms of mechanism function, respectively. Substitute $\alpha_{i}$, $T_{i}$, and ${\left[d\alpha /dt\right]}_{i}$ obtained during the experimental process in equation (2.8) to obtain the experimental curve


(2.8) $$y\left(\alpha \right)={\left(\frac{T_{i}}{T_{0.5}}\right)}^{2}\cdot \frac{\left[d\alpha /dt\right]}{{\left[d\alpha /dt\right]}_{0.5}},$$

where $T_{i}$ and [dα/dt] represent reaction temperature and rate corresponding to the conversion rate of α. The highest degree of coincidence with the standard curve is the most probable mechanism function [38]. A detailed analysis method can be found in the previous studies [39].

The collected particulate matter was weighed using a microbalance with an accuracy of 0.001 mg to calculate the particle size distribution. Each set of experiments was done at least three times to ensure repeatability. The standard deviation of experimental data was calculated using the STDEV function in Excel software. The particulate matter samples were carbon-sprayed. Thereafter, the elemental composition of particulate matter in different regions was analysed using backscatter electron scanning electron microscope. The elemental content of particulate matter was averaged. A detailed description of the test methods can be found in previous studies [40].

## 3. Results

### 3.1. Combustion characteristic analysis

**Figure 1.**
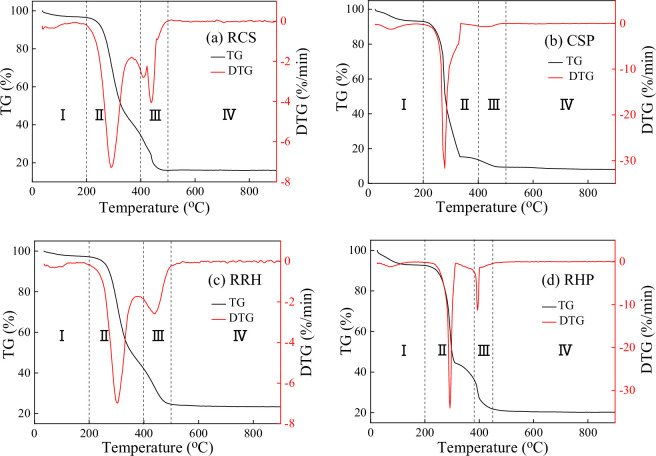
TG and DTG curves of raw biomass and biomass pellets.

TG-DTG curves of all samples are presented in figure 1. By combining weight loss and weight loss rate curves, the whole combustion process can be divided into four stages. For RCS, CSP and RRH, the temperatures corresponding to the four stages were 0−200°C (stage I), 200−400°C (stage II), 400−500°C (stage III) and 500−900°C (stage IV), respectively. The temperatures at the four combustion stages of RHP, were slightly different: 0−200°C (stage I), 200−380°C (stage II), 380−450°C (stage III) and 450−900°C (stage IV). The weight loss at stage I was mainly caused by evaporation of water, whereas the weight loss at stage II was caused by the release and combustion of volatile matter. The third stage mainly involved the combustion of fixed carbon and probably a small amount of volatile combustion. The weight loss at stage IV was relatively small for all samples, due to the decomposition of mineral content [41]. It can be seen that stages II and III were the main combustion stages, and the total weight loss exceeded 70%, among which the CSP weight loss was the highest, reaching 83.55%.

The weight loss curves of raw biomass and biomass pellets were quite different, as shown in figure 1a–d. The DTG curves of biomass pellets were steeper, indicating a more concentrated and intense combustion process. In the case of CSP, the combustion of volatile matter and fixed carbon almost completely overlapped, with only one prominent weight loss peak. Comparing figure 1a,c, a shoulder peak at approximately 420°C was observed for RCS, probably due to higher volatile content in RCS than in other biomass samples. This indicates that stage III of RCS included the combustion of residual volatiles and fixed carbon.

The characteristic parameters of raw biomass and biomass pellets can be seen in table 3. The ignition temperature, burnout temperature, DTG_max_, DTG_mean_, ignition index and burnout index of RCS were higher than those of RRH, indicating that RCS were more prone to combustion. In general, the flammability and composite combustion indices of RCS were higher than those of RRH, indicating the superiority of RCS combustion performance. Compared with RCS, ignition and burnout temperature of CSP remained unchanged, but the maximum combustion temperature decreased. The increase in DTG_max_ and DTG_mean_ further indicated that CSP combustion was concentrated and intense. The ignition, burnout, flammability and composite combustion indices of cotton stalk increased significantly, indicating that the combustion performance was improved by densification pretreatment. Compared with that of RCS and CSP, the combustion of RHP was much more intense than that of RRH, with greater amplification of ignition, burnout, flammability and composite combustion indices, indicating that densification pretreatment significantly improved the combustion characteristics of rice husk.

**Table 3.** The characteristic parameters of the biomass.

samples	*T*_i_ (^o^C)	*T*_b_ (^o^C)	DTG_max_ (%/min)	*T*_max_ (^o^C)	DTG_mean_ (%/min)	*D*_i_ × 10^−3^	*D*_b_ × 10^−4^	*C* × 10^8^	*S* × 10^13^
RCS	258	457	7.2842	289	0.9685	12.84	10.34	25.83	34.27
CSP	259	462	31.6702	278	0.9409	47.80	149.20	111.90	143.25
RRH	268	489	6.9787	302	0.8839	11.10	9.50	23.84	27.66
RHP	273	448	34.0742	292	0.8176	47.29	358.52	114.30	129.61

C, flammability index; D_b_, burnout index; D_i_, ignition index; DTG_max_, maximum combustion rate; S, composite combustion index; *T*_b_, burnout temperature; *T*_i_, ignition temperature; *T*_max_, corresponding temperature of the maximum combustion rate.

### 3.2. Combustion kinetics analysis

Stages II and III were the main combustion zones. The combustion reaction kinetics of raw biomass and biomass pellets are shown in table 4. The main combustion zone of RCS can be divided into three stages, corresponding to 200−365, 265−435 and 435−457°C. The stage 1 and stage 3 were the reaction series models (F3/2 and F2), whereas stage 2 was the three-dimensional diffusion mechanism model (D3). Similar to RCS, the main combustion zone of CSP can be divided into three stages, with narrow temperature range, which conforms to one-dimensional diffusion mechanism models (D1), reaction series models (F3/2) and three-dimensional diffusion mechanism models (D3). The activation energy of the CSP in the first two stages was higher than that of RCS, indicating that the difficulty of initial combustion increased after densification pretreatment. However, the activation energy of CSP at stage 3 was only 13.98 kJ mol^−1^, which was significantly lower than that of the RCS (313.62 kJ mol^−1^), indicating that the densification pretreatment improved the combustion at this stage.

**Table 4.** Combustion reaction kinetics results of the biomass.

samples	stages	temperature (℃)	activation energy (kJ mol^−1^)	pre-exponential factor	correlation coefficient	models
RCS	1	200−365	57.40	17 981	0.9672	F3/2
	2	365−435	40.30	23.68	0.9784	D3
	3	435−457	313.62	9.38e + 23	0.9876	F2
CSP	1	220−278	94.40	2.74e + 07	0.9633	D1
	2	278−333	72.58	1.98e + 06	0.9912	F3/2
	3	400−462	13.98	0.1619	0.9908	D3
rice husk	1	200−380	107.97	3.11e + 07	0.9618	D3
	2	380−489	50.97	178.91	0.9844	D3
rice husk pellet	1	230−310	114.89	3.39e + 09	0.9511	D1
	2	335−388	20.13	0.3888	0.9346	D3
	3	388−400	170.76	3.91e + 13	0.9968	F3/2
	4	400−448	64.81	9.03e + 04	0.9998	F3/2

Unlike RCS, the main combustion zones of RRH consisted of stages 1 (200−380℃) and 2 (380−489℃), both of which were three-dimensional diffusion mechanism models (D3). Overall, the activation energy of RRH in the main combustion area was higher than that of RCS, indicating that the combustion performance of RRH was relatively poor. The combustion of RHP was much more complex and can be divided into four stages, corresponding to 230−310, 335−388, 388−400 and 400−448°C. Compared with RRH, the oxygen diffusion capacity of RHP at the initial combustion stage was weaker due to high density and low porosity. Therefore, the activation energy at stage 1 was 114.89 kJ mol^−1^ and belonged to the one-dimensional diffusion mechanism model (D1). At stage 2, the oxygen diffusion capacity increased, activation energy decreased rapidly and the reaction mechanism shifted to the three-dimensional diffusion mechanism model (D3). At stage 3, the RHP char began to burn. Due to high ash content and large proportion of non-combustible silica, activation energy increased again, which was the reaction series model (F3/2). The combustion activation energy at stage 4 decreased to 64.81 kJ mol^−1^ and the combustion performance improved, which was still the reaction series model (F3/2). Compared with that of cotton stalk, densification pretreatment had a greater impact on the combustion of rice husk.

### 3.3. Particulate matter emissions

**Figure 2.**
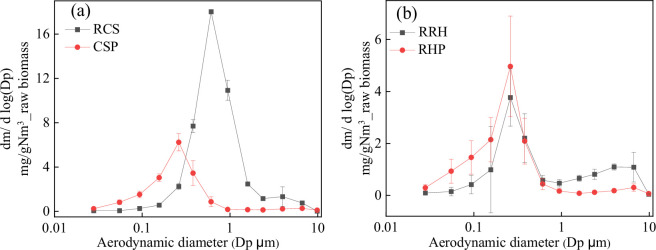
Particle size distributions (PSDs) of PM_10_.

The particle size distributions (PSDs) of PM_10_ from raw biomass and biomass pellets can be seen in figure 2. Figure 2a shows a bimodal distribution of PM during RCS combustion, with a peak of fine particle mode around 0.6 μm and a peak of coarse particle mode around 3.97 μm. Table 5 shows the yield of PM with different aerodynamic diameters. The ratio of PM_1_ to PM_10_ was 87.41%. Therefore, PM_10_ was mainly composed of fine particles smaller than 1 μm. Compared with that of RCS, the PSDs of CSP exhibited unimodal distribution, and the peak shifted forward to approximately 0.26 μm. Table 5 shows that the yield of PM_10_ and PM_1_ decreased from 45.58 and 39.84 to 15.75 and 14.85 mg Nm^−3^, respectively, which indicated that densification pretreatment was beneficial for reducing PM emission. However, the yield of PM_0.1_ and the ratio of PM_1_ to PM_10_ increased from 0.38 mg Nm^−3^ and 87.41% to 2.61 mg Nm^−3^ and 94.29%, respectively, indicating that densification pretreatment was conducive to reduce the emission of PM_0.1–10_, but has no obvious inhibitory effect on ultrafine particles (<0.1 μm).

**Table 5.** Yields of PM with different aerodynamic diameters.

samples	PM_0.1_	PM_0.1–1_	PM_1_	PM_1–10_	PM_10_	ratio of PM_1_ to PM_10_, [%]
	[mg Nm^−3^]					
RCS	0.38 ± 0.03	39.46 ± 0.08	39.84 ± 0.05	5.74 ± 0.35	45.58 ± 0.40	87.41
CSP	2.61 ± 0.55	12.24 ± 0.55	14.85 ± 3.68	0.90 ± 0.05	15.75 ± 3.63	94.29
RRH	0.66 ± 0.16	8.01 ± 1.55	8.67 ± 1.60	3.70 ± 0.30	12.37 ± 1.89	70.09
RHP	1.81 ± 0.31	7.19 ± 0.97	8.99 ± 1.22	0.73 ± 0.27	9.72 ± 1.08	92.48

PM_0.1_, the yield of particles of aerodynamic cut-off diameters less than 0.1 μm; PM_1_ , the yield of particles of aerodynamic cut-off diameters less than 1 μm; PM_10_, the yield of particles of aerodynamic cut-off diameters less than 10 μm; PM_0.1–1_, the yield of particles of aerodynamic cut-off diameters between 0.1–1 μm; PM_1–10_, the yield of particles of aerodynamic cut-off diameters between 1–10 μm.

Figure 2b shows that the PSDs of RRH appeared in a bimodal distribution, with the peaks of fine and particle coarse modes at approximately 0.26 and 6.65 μm, respectively. As shown in table 5, the PM_10_ yield of RRH (12.37 mg Nm^−3^) was significantly lower than that of RCS (45.58 mg Nm^−3^), but the PM_0.1_ yield was the opposite. The ratio of PM_1_ to PM_10_ of RRH was 70.09%, indicating that although PM_10_ was mainly composed of fine particles, the coarse particles could not be ignored. Compared with those of RRH, the PSDs of RHP still exhibited a bimodal distribution, and the peak remained unchanged. As shown in table 4, PM_10_ yield of RHP decreased to 9.72 mg Nm^−3^, but the decline was significantly lower than that of cotton stalk. The yields of PM_0.1_, PM_1_ and the ratio of PM_1_ to PM_10_ increased from 0.66 mg Nm^−3^, 8.67 mg Nm^−3^ and 70.09% to 1.81 mg Nm^−3^, 8.99 mg Nm^−3^ and 92.48%, respectively, indicating that densification pretreatment mainly reduced coarse particle emission but increased fine particle formation. In general, densification pretreatment is beneficial to reduce particulate matter emission from agricultural biomass, and the inhibitory effect on fine particles, especially ultrafine particles, is not obvious or even promoted. This should be related to the combustion process, and the details will be further discussed in §3.5.

### 3.4. Particulate matter composition

**Figure 3.**
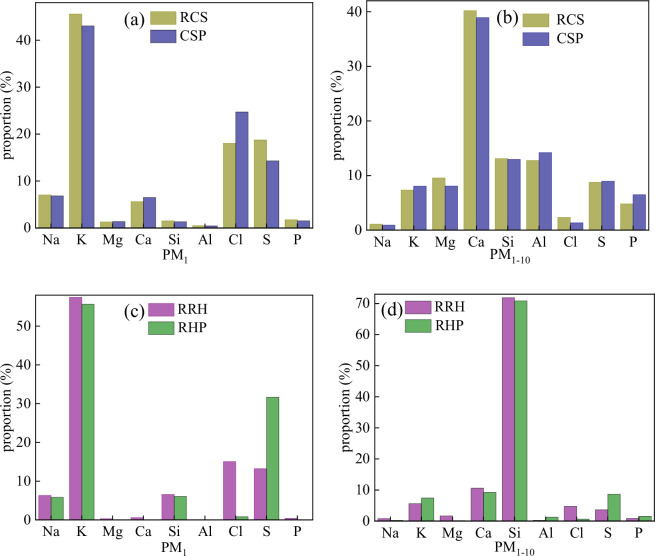
Elemental composition of PM.

Figure 3 shows that PM was mainly composed of PM_1_ and PM_1–10_, therefore, the components of these two PMs were analysed in detail. As seen in figure 3a, the PM_1_ of RCS was mainly composed of K, Cl, S, Na and Ca, with a small amount of Mg, Si, Al and P. The molar ratio between the main elements in PM_1_ is shown in table 6. The molar ratio of (Na + K)/(Cl + 2S) was close to 1, indicating that alkali metals mainly existed as sulfides and chlorides. The molar ratio of (Na + K)/Cl was higher than that of (Na + K)/2/S, indicating that the alkali metal sulfides content was higher than that of alkali metal chlorides. Alkaline earth metals were relatively low and probably existed in the form of oxides or carbonates [42]. More experimental evidence is needed. Figure 3b shows that the PM_1–10_ of RCS was mainly composed of Ca, Si, Cl, Mg, S, K and P, arranged in a decreasing order of content. As shown in figure 3a,b, densification pretreatment did not change the composition of PM_1_ and PM_1–10_, but affected the content of each element. For PM_1_, densification pretreatment increased the content of Ca, Cl and Mg, but decreased the content of K, S and Na. This could be due to the difference in the mechanism of particulate formation between alkali and alkaline earth metals. Early studies have shown that alkali metals are volatile and form PM_1_ through vaporization−release–condensation during combustion [40]. Densification pretreatment inhibited the release of ash and impeded the gasification of alkali metals in ash, thus reducing the conversion of alkali metals to PM_1_ [43]. Alkaline earth metals get directly converted into submicrometre particles through heterogeneous condensation or react with Si/P to form PM_1_ [44]. The impact of densification pretreatment on alkaline earth metals was relatively weak, therefore, their relative content increased. The decrease in K and S content indicated that densification pretreatment was beneficial for inhibiting the transformation of alkali metal sulfides into particulate matter. For PM_1–10_, the influence of densification pretreatment on the content of each element was not obvious.

**Table 6.** The molar ratio between the main elements in PM_1_.

molar ratio	RCS	CSP	RRH	RHP
(Na + K)/Cl	2.92	2.78	4.23	74.55
(Na + K)/2 /S	1.40	1.51	2.41	0.97
(Na + K)/(Cl + 2S)	0.95	0.98	1.54	0.96

Figure 3c shows that the PM_1_ of RRH was mainly composed of alkali metals, Si, Cl and S. As shown in table 6, the molar ratio of (Na + K)/(Cl + 2S) was greater than 1, indicating that alkali metals form polymers with Si in addition to chloride and sulfate. As shown in figure 3d, PM_1–10_ was mainly composed of Si, Ca and K, indicating that silicate is the main component. For PM_1_, densification pretreatment reduced the content of alkali metals, Cl and Si, unlike cotton stalk. The previous analysis indicated that densification pretreatment improved combustion characteristics. Moreover, rapidly released volatile alkali metal compounds could not effectively contact and react with silicon-containing compounds, resulting in an increase in the conversion rate of alkali metals to particulate matter, thus increasing the yield of PM_1_ [45]. Similar to cotton stalk, densification pretreatment had a weaker effect on the PM_1–10_ component content.

## 4. Discussion

The influence of densification pretreatment on biomass combustion and particulate matter emissions from the two types of agricultural biomass is evident. The sample characteristics affect the influence mechanism of pretreatment, which is further discussed.

For the two biomass samples, densification pretreatment strengthened the contact degree of internal particles, resulting in overlapping of volatile matter and char combustion, as shown in figure 1. The effect of densification pretreatment on the DTG curve of cotton stalk was greater than that of rice husk, with only one prominent weight loss peak. This could be due to the high volatility and low ash content of cotton stalk. The release and combustion time of cotton stalk volatile components were relatively long, overlapping with char combustion and ultimately presenting a major weight loss peak. Zha *et al*. [46] demonstrated that the ash shell generated by the surface combustion of biomass particles would hinder oxygen diffusion and delay char combustion. The high ash content of rice husk enhanced this effect, resulting in two major peaks in the DTG curve.

For cotton stalks, the diffusion mechanism models were dominant after densification pretreatment, and the activation energy of char combustion decreased. The alkali metals in ash had a catalytic effect on char combustion, and densification pretreatment promoted the contact between ash and char, which strengthened this effect [47]. For rice husk, densification pretreatment converted the combustion mechanism model of char from diffusion mechanism to reaction series model and increased activation energy. It can be explained as follows: (i) there were few alkali metals in ash, and the catalytic effect was weak [48], (ii) the non-combustible silicon hindered heat transfer, and (iii) the endothermic reaction of ash further weakened the catalytic effect of alkali metals [49].

As for particulate matter emissions, densification pretreatment mainly reduced the PM_0.1–1_ yield of cotton stalk and the PM_1–10_ yield of rice husk. Elemental composition analysis showed that densification pretreatment did not change the composition of PM_1_ and PM_1–10_, but affected the content of each element. For cotton stalk and rice husk, densification increased the release of fine particles (PM_0.1_) as shown in table 5, which could be due to the combustion. Densification resulted in concentrated and intense combustion, which promoted the rapid release of small amounts of alkali metals, resulting in the formation of fine particulate matter (PM_0.1_). For cotton stalk, the decrease in K and S content indicated that densification pretreatment is beneficial for inhibiting the transformation of alkali metal sulfides into PM_0.1–1_. However, the influence of densification pretreatment on the content of each element of PM_1–10_ was not obvious. As shown in figure 3d, Si is dominant in PM_1–10_ of rice husk. Early studies have shown that silicon can be converted into PM_1–10_ through two pathways: (i) Si can be oxidized into small particles of silica dioxide (1–10 µm) during combustion [50], and (ii) silicon dioxide can react with alkaline earth metals at high temperature to form alkaline metal silicates, which then undergo a series of physical/chemical reactions to form PM_1–10_ [51]. The volatility of Si-containing compounds is poor, and densification pretreatment inhibits the release and reaction of Si-containing compounds with other elements in rice husk, resulting in a decrease in PM_1–10_ generation.

The effect of densification on combustion characteristics is closely related to the content of volatile matter, alkali metals and ash. Volatile and alkali metals play positive roles, whereas ash plays the opposite in combustion. For biomass densification and combustion, biomass with high volatile matter, high alkali metals and low ash content is preferred. However, high content of alkali metals can promote the formation of particulate matter, especially fine particulate matter (PM_1_). The removal of fine particulate matter is difficult and undesirable. Overall, to ensure good combustion efficiency and low levels of particulate matter emissions, biomass with high volatile matter, low alkali metal and ash contents is the optimal choice.

## 5. Conclusions

By analysing the combustion and particulate matter emission characteristics of raw biomass and biomass pellets, the influence of densification pretreatment was clarified, and the conclusions of this study are as follows:

1. Densification pretreatment improves the combustion performance, resulting in concentrated and intense combustion. However, the combustion models become complicated due to densification pretreatment.
2. For cotton stalks, densification pretreatment helps reduce the emission of PM_0.1–10_, but has no obvious inhibitory effect on ultrafine particles (<0.1 μm). Densification pretreatment is beneficial for inhibiting the transformation of alkali metal sulfides into particulate matter.
3. For rice husk, densification pretreatment mainly reduces coarse particle (PM_1–10_) emission but increases the formation of fine particles (PM_1_). The rapidly released volatile alkali metal compounds cannot effectively contact and react with silicon-containing compounds, resulting in an increase in the conversion rate of alkali metals to particulate matter, thus increasing the yield of PM_1._
4. For PM_1–10_, the influence of densification pretreatment on the content of each element is unnoticeable in both biomass samples.

## Data Availability

The data that support the findings of this study are openly available in Dryad [52].
